# Acoustic Waves and Their Application in Modern Fire Detection Using Artificial Vision Systems: A Review

**DOI:** 10.3390/s25030935

**Published:** 2025-02-04

**Authors:** Jacek Lukasz Wilk-Jakubowski, Valentyna Loboichenko, Mikhail Divizinyuk, Grzegorz Wilk-Jakubowski, Roman Shevchenko, Stefan Ivanov, Viktor Strelets

**Affiliations:** 1 Department of Information Systems, Kielce University of Technology, 7 Tysiąclecia Państwa Polskiego Ave., 25-314 Kielce, Poland; 2 Departamento de Ingeniería Energética, Escuela Técnica Superior de Ingeniería, Universidad de Sevilla, Camino de los Descubrimientos s/n., 41092 Sevilla, Spain; vloboichm@gmail.com; 3 Department of Civil Security, Lutsk National Technical University, Lvivska St., 75, 43000 Lutsk, Ukraine; 4 Center for Information–analytical and Technical Support of Nuclear Power Facilities Monitoring of the National Academy of Sciences of Ukraine, 34A, Academician Palladin Ave, 03142 Kyiv, Ukraine; divizinyuk@ukr.net; 5 Institute of Internal Security, Old Polish University of Applied Sciences, 49 Ponurego Piwnika Str., 25-666 Kielce, Poland; grzegorzwilkjakubowski@wp.pl; 6 Institute of Crisis Management and Computer Modelling, 28-100 Busko-Zdrój, Poland; 7 Science and Innovation Center, National University of Civil Defence of Ukraine, 94 Chernyshevska Str., 61023 Kharkiv, Ukraine; shevchenko605@i.ua; 8 Department of Automation, Information and Control Systems, Technical University of Gabrovo, Hadji Dimitar 4, 5300 Gabrovo, Bulgaria; st_ivanov@abv.bg; 9 Institute of Public Administration and Research in Civil Protection, Vyshhorodska St., 21, 04074 Kyiv, Ukraine; vstrelec1956@ukr.net

**Keywords:** security, acoustic wave, mathematical model, acoustic extinguisher, computer vision, data processing, electrical engineering, machine learning, fire safety

## Abstract

This paper presents information on the propagation patterns of acoustic waves and their practical application, in particular, in modern fire detection methods that use artificial vision systems and video cameras as intelligent sensors. In practice, the use of artificial intelligence allows the detection of flames in places where the use of typical sensors is impossible or severely limited. Such a system can work together with environmentally friendly acoustic flame extinguishing technology as a standalone platform, or it may cooperate with other modules, which is a new approach in the field of fire protection. An analysis shows that the presented eco-friendly methods outperform other methods, with many advantages. In the future, the acoustic method can be used for the monitoring and early detection of fires in factory buildings or objects of high cultural, religious, and historical value, while an acoustic extinguisher equipped with artificial vision systems can be successfully used to extinguish fires.

## 1. Introduction

Fires are among the dangerous natural and man-made phenomena that humankind has faced throughout its existence. Large-scale forest fires, disruption, and the destruction of urban infrastructure caused by flames lead to economic losses and, often, loss of life [1]. Fires themselves can be primary or secondary in nature, being the main source of danger or the result of another emergency situation, but their result, in any case, is a negative impact on people and the environment [2,3,4]. The known methods of extinguishing fires involve the use of extinguishing agents such as water, gasses, and foams of various compositions. It should be noted that the fire class (A, B, C, D, K, or F) [5,6] affects the fire extinguishing means used for the elimination of fires. In recent years, the orientation of developed countries towards compliance with sustainable development goals has led to the search for environmentally safe fire extinguishing agents, such as fluorine-free fire extinguishing foams, silicon-based foaming agents, gels, and environmentally safe water additives [7,8,9,10,11,12]. The search for environmentally safe materials (fire retardants) is underway [13,14] to ensure the safety of people. Robotic firefighting systems are being developed [15], and their evacuation methods are being optimized [16]. One of the important methods of minimizing the negative impact of fires is to prevent their development with subsequent (if necessary) elimination in early stages. In this direction, the use of acoustic effects in firefighting can be noted. To ensure the acoustic extinguishment of a flame, the location of the sound source close to the flame, standing waves in the premises to extinguish the flame, and the focusing of high-amplitude sound at a distance are used [17]. The possibility of the low-frequency acoustic extinguishing of fires of various organic substances, such as ethanol, has been claimed [18,19]. It may also be possible to use this method for extinguishing fires involving gasoline, kerosene, thinner, liquefied petroleum gas [20], wood, plasterboard, paper, motor oil, and paraffin [21], and there is the possibility of orientation in a smoky room using this method [21] and the identification of the type of fire using ultrasound [22]. The influence of a low-frequency sound stream (200–300 Hz) on flame morphology has been shown [23,24,25], and a criterion for the attenuation of a jet diffusion flame has been proposed [26]. Authors have discussed the need for further research into the features of low-frequency acoustic fire extinguishers to ensure safety and fire extinguishing efficiency [27]. The advantages of using this method in the early stages of fire extinguishing [20,27], when dripping molten material (polyethylene) [28], and when extinguishing small and medium fires [29], as well as the effectiveness of its application when extinguishing fires in space [30], have been observed. The perspective of using the acoustic method in the location of fires in natural ecosystems has been shown, in particular, in the extinguishing of flying sparks and firebrands [31]. In [32,33], machine learning approaches are used to predict the possibility of acoustic fire extinguishing with given parameters. It is proposed to combine the possibilities of the early detection of fires with the help of deep neural networks (on an artificial intelligence platform) with acoustic fire extinguishing, combined into a single fire protection system [34], including the use of mobile robots [35,36,37].

This research focuses on the theoretical features of sound wave propagation and the possibility of using modern imaging techniques to detect flames. This is particularly important when detecting flames in open spaces (traditional sensors have a limited operating range and, therefore, are not usually used to detect flames in open spaces). Moreover, classical sensors are also limited due to environmental conditions. Here is where artificial intelligence comes to aid. When classical sensors are used, there is a sharp drop in efficiency in wide geographical areas. Therefore, video-based fire detection is the most suitable for detecting fires in open spaces outside buildings, i.e., forests, where fire can be spotted up to 10 km away. It should be emphasized that in this type of application (forests), it is not possible to use fire detection methods based on computer vision together with acoustic technology, which is in an early stage of development (the range of acoustic technology is much shorter; at present, it is a few meters) [36,37,38,39,40,41,42,43,44]. However, an undoubted advantage of systems based on image processing using computer techniques is that they can be applied not only to outdoor environments over large geographical areas, but also to indoor environments. In contrast to classical sensors, changes in the environment and ambient conditions generally do not affect their operation. In addition, the use of computer vision-based fire detection methods is also economically justified (low-cost sensors). For these reasons, these techniques are increasingly being used.

In this article, the analyses carried out concern, among other things, a review of the state of the art in current flame detection techniques. For this purpose, the mathematical laws of acoustic wave propagation are considered, and an overview of fire detection techniques and properties for fire detection purposes is provided, amongst other things. A comparison of modern fire detection techniques using artificial vision systems and video cameras, including fire detection categories, is provided. In this sense, based on the mathematical laws and literature review method, the authors present a mathematical model of acoustic temperature control in an isolated room, the state of the art in this topic based on the latest research, combining both parts (concise review of computer vision flame detection techniques and flame extinguishing after flame detection). Consequently, the aim of this paper is to theoretically study the laws of acoustic wave propagation in a room and the factors influencing it, as well as to propose an extinguishing system with camera-based fire detection and an acoustic extinguishing module. Practical research in this field is carried out under European cooperation. As indicated above, the possibility of equipping the acoustic extinguisher with an intelligent module is a scientific novelty (such systems can work together). The authors emphasize the development of algorithms for the control of autonomous acoustic fire extinguishers. They present the possibility of flame detection (considering currently known techniques and algorithms) and flame extinguishing using novel environmentally friendly methods (including the acoustic technique) [45,46,47,48]. However, acoustic waves also have many other applications [49,50,51].

The main contribution of the authors is the mathematical model of acoustic temperature control in an isolated room, and the detection of fires and outbreaks by means of pulse acoustic probing, as well as the combination of an acoustic high-power fire extinguisher with modern approaches to the use of deep neural networks. In this regard, the article also includes a review of studies by other researchers. The solutions are evaluated and compared to other applied methods in terms of their benefits. The information presented seems to be essential, strong, and helpful in the development of an eco-friendly and safe concept for human acoustic firefighting. An intention of the authors in this aspect is to develop a modern autonomous acoustic fire extinguisher that uses artificial intelligence and can autonomously take decisions.

## 2. Mathematical Laws of Acoustic Wave Propagation

### 2.1. The Pattern of Decrease in the Intensity of a Propagating Acoustic Wave

In direct measurements, the value of the speed of sound (in continuous media such as liquids and gasses [52,53]) is obtained directly during the measurements, and when using indirect methods, it is calculated using known formulas in which the values of quantities obtained by direct measurements are substituted. These quantities are functionally related to the calculated speed of sound. For the atmosphere, this is air temperature, humidity, and atmospheric pressure. An obvious advantage of direct methods is that the measurement result not only immediately gives the speed value, but also allows for a qualitative assessment of the change in primary parameters, such as air temperature. Regardless of which direct methods of measuring the speed of sound are used, their essence lies in the emission of an acoustic wave and the subsequent reception of its reflection from a reflector installed at the opposite end of a fixed measuring base. The energy characteristic of direct measurements is determined by the laws of acoustic wave propagation using the parameters of the emitter and receiver of sound, as well as the reflector [54]. The optimal detection of a reflected signal is based on the excess of the level or intensity of the received useful signal $I_{sig}$ over the level of interference $I_{int}$ arriving at the input of the receiving device [55,56]; that is, the following condition must be met:

(1) $$I_{sig}\geq \delta \cdot I_{int}=\delta \cdot \frac{P_{rd}}{K_{da}}\;,$$

where δ is the recognition coefficient (a dimensionless value that depends on the signal processing methods in the receiving acoustic device).

The right side of the equation is determined by the sensitivity of the receiving device—$P_{rd}$. This value is reduced by the directional reception of the acoustic receiver by an amount equal to the coefficient of its directional action—$K_{da}$. The left side of Equation (1) is determined by the power—$P_{em}\;$—of the acoustic signal emitted into space. This can be the power of the acoustic pulse (in pulse scanning) or the average radiation power in continuous acoustic radiation. The sound wave propagates according to the spherical law. Its front expands, and the intensity of the wave decreases proportionally to the square of the current distance, that is, proportionally ${\left(4\pi D\right)}^{2}$. Since the acoustic emitter has a directivity characterized by the coefficient of directional action—$K_{da}$, the current value of the intensity of the acoustic wave will be increased by an amount equal to this coefficient.

Propagating in air space, the intensity of the acoustic wave decreases not only due to the expansion of the wave front, but also due to the volume attenuation, the value of which is determined by the volume attenuation coefficient *β*. Its value depends on the frequency of acoustic oscillations propagating in space, and is found empirically. Therefore, the current value of the sound intensity will be as follows:

(2) $$\frac{P_{em}\cdot K_{da}}{{\left(4\pi D\right)}^{2}}\cdot 10^{-0.1\cdot \beta D_{km}}.$$

A reflector irradiated by an acoustic wave reflects only a portion of the energy falling on it, which is determined by the area of the reflecting surface. It will be equal to $2\pi {R_{r}}^{2}$, where $R_{r}$ is the reflector radius. This is the portion of acoustic energy that will be reflected and propagated in the opposite direction.

The reflected wave propagating in the opposite direction will be attenuated due to the expansion of the wave front proportional to the square of the current distance—(4π*D*)^2^. Its intensity value will decrease due to volumetric attenuation by the value $10^{-0,1\cdot \beta D_{km}}$ (expressed in dB/km).

Then, the intensity of the acoustic wave reflected from the reflector will take the following form:

(3) $$\frac{P_{em}\cdot K_{da}}{{\left(4\pi D\right)}^{2}}\cdot 10^{-0.1\cdot \beta \cdot D_{km}}\cdot 2\pi \cdot R_{r}^{2}\cdot \frac{10^{-0.1\cdot \beta \cdot D_{km}}}{{\left(4\pi D\right)}^{2}}\;.$$

After performing the transformations, substituting (3) into (1), we obtain the following expression:

(4) $$\frac{P_{em}\cdot K_{da}\cdot 2\pi \cdot R_{r}^{2}}{{\left(4\pi D\right)}^{4}}\cdot 10^{-0.2\cdot \beta \cdot D_{km}}\geq \delta \cdot \frac{P_{rd}}{K_{da}}\;.$$

Taking the logarithm of inequality (4) and performing the transformations (let one perform the so-called translation in decibel form), we obtain the following:

(5) $$40\cdot \mathrm{lg}4\pi +10\cdot \mathrm{lg}\left(2\pi \right)+40\cdot \mathrm{lg}D+2\beta D_{km}\;\leq 10\cdot \mathrm{lg}\delta +10\cdot \mathrm{lg}P_{rd}-20\cdot \mathrm{lg}K_{da}-10\cdot \mathrm{lg}P_{em}-20\cdot \mathrm{lg}R_{r}.$$

Based on the fact that the number π is a constant, we denote the sum ($40\cdot \mathrm{lg}4\pi +10\cdot \mathrm{lg}\left(2\pi \right)$) through 2*K*. After transformations, we finally obtain the following:

(6) $$20\cdot \mathrm{lg}D+\beta D_{km}+K\;\leq \frac{1}{2}\left(10\cdot \mathrm{lg}\delta +10\cdot \mathrm{lg}P_{rd}-20\cdot \mathrm{lg}K_{da}-10\cdot \mathrm{lg}P_{em}-20\cdot \mathrm{lg}R_{r}\right),$$

where K ≈ 10.98 dB.

Expression (6) is commonly called a non-strict inequality of the acoustic detection range. Its left-hand side is called the regularity of the decay of the intensity of the propagating acoustic wave, and is designated as $\Psi \left(D,f\right)$. On its right side, there are five terms, expressing, in decibel form, the values of the four main technical parameters of the acoustic device, namely the following: the recognition coefficient *δ*, the sensitivity of the receiving device $P_{rd}$, the directional coefficient $K_{da}$, the radiation power $P_{em}$, and the radius of the reflector $R_{r}$. This part of the inequality is denoted as $E_{ad}$—the energy potential of the acoustic device. Now, expression (6) will take the following form:

(7) $$\Psi \left(D,f\right)\leq \frac{1}{2}E_{ad}.$$

Replacing, on the left side, the length of the acoustic base *L* (the acoustic base is the distance between the emitter-receiver and the reflector) in expression (6) with the current value of the distance *D*, and on the right side, denoting the energy potential of the acoustic device as a functional dependence on the pulse power of the acoustic device, we obtain (8):

(8) $$\Psi \left(L,f\right)\leq \frac{1}{2}E_{ad}\left(P_{imp}\right).$$

Consequently, the use of pulse (discrete) scanning in acoustic devices allows the monitoring of the condition of large internal spaces without increasing the electrical power consumption of the facility.

### 2.2. The Influence of Temperature Variability in the Indoor Air Environment, as a Result of Season and Day Changes, on the Change in the Speed of Sound

When studying the acoustic properties of the atmosphere, ensuring control of the state of the air environment in a local room, dependence (9) [57] was obtained, determining the change in the speed of sound from the change in the primary parameters of the air environment:

(9) $$C=\sqrt{\gamma \frac{RT}{M}}.$$

where γ is the adiabatic index (1.4 for a normal atmosphere); *R* is the gas constant (8.314 J/(mol·K)); *M* is the molar mass; and *T* is the temperature, K (K = 273.15° + T °C).

Assuming that the primary parameters of the air environment, with the exception of temperature, remain unchanged, expression (9) allows us to obtain a functional dependence of the speed of sound on air temperature:

(10) $$C=\phi \left(T^{\prime }C\right).$$

Under these same assumptions, the second functional dependence (11) is also valid, allowing us to obtain the temperature value from the measured values of the speed of sound in the atmosphere:

(11) $$T^{\prime }C=f\left(C\right).$$

Since, in the measuring acoustic device, the speed of sound is measured by the time $t_{i}$ of the passage of the acoustic wave along the measuring base *L* to the reflector and back, the temperature in the room $T^{\prime }C$ will be determined by the set of n measurements performed (12):

(12) $$T^{\prime }C=\varphi \left(\frac{1}{n}\sum_{i=1}^{n}C_{i}\left(L,t_{i}\right)\right).$$

### 2.3. The Mathematical Model of Acoustic Temperature Control in an Isolated Room, and the Detection of Fires and Outbreaks by Means of Pulse Acoustic Probing

It is expected that the mathematical model of acoustic temperature control in an isolated room, and the detection of fires and outbreaks inside it by means of pulsed acoustic probing, should solve a number of tasks. Firstly, the task of pulse probing of the entire volume of the room. Secondly, measuring the air temperature in the entire space of the room. Thirdly, detecting fires and conflagrations inside the room. The fourth task is to take into account the dimensions (volume) of the room when detecting fires.

The first task is solved by using dependence (8), which is the functional dependence of the length of the acoustic base *L* on the pulse power of the acoustic device. This dependence allows, depending on the size of the rooms, the calculation and determination of the main technical parameters of the designed acoustic devices. Conversely, having a set of acoustic devices or technical characteristics of electronic elements, it is possible to calculate the size of rooms that can be served by these devices.

The solution to the second task is provided by expression (12), which, based on the time *t_i_* of the passage of the acoustic wave along the measuring base *L* to the reflector and back, allows the determination of the average air temperature in the room, based on the totality of the measurements taken. The value *n* is determined by the number of reflectors located along the perimeter of the room.

The first and third acoustic paths go along the walls of the room. The fifth acoustic path goes diagonally across the room. The second and fourth tracks are located between them, ensuring complete coverage of the entire room. Any increase in temperature in the local volume will lead to an increase in the speed of sound and a decrease in the signal travel time along the corresponding acoustic track. This in turn allows us to fix the expression (12).

To solve the third task, it is necessary to establish threshold values for the air temperature in the room $T_{\;thr}^{\prime }$ (for example, 50 °C or 60 °C) and the value of the temperature increase per second (time gradient) $\Delta T_{thr}^{\prime }$ (for example, 0.25 degrees/s); exceeding these indicates the beginning of the combustion process. The formalized mathematical record of the solution to this problem will look like system (13):

(13) $$\left\{\begin{array}{cc} \Delta T_{j+1}^{\prime }=T_{j+1}^{\prime }-T_{j}^{\prime } & \\ \Delta T_{j+2}^{\prime }=T_{j+2}^{\prime }-T_{j+1}^{\prime } & T_{j+l}^{\prime }>T_{\;thr}^{\prime }\\ \ldots \ldots \ldots \ldots & \Delta T_{j+l}^{\prime }>\Delta T_{thr}^{\prime }\\ \Delta T_{j+l}^{\prime }=T_{j+l}^{\prime }-T_{j+l-1}^{\prime } & \end{array}\right.$$

where *l* is the number of successively emitted probing acoustic pulses, according to the results of which the reception of the reflected signals shows that the temperature threshold has been exceeded and the rate of its growth has exceeded the threshold value.

The number *l* can be chosen in accordance with the likelihood criteria, for example, three excesses out of three, or four out of four, or five out of five, and others.

Combining dependencies (8), (12), and system (13) into one system, we obtain the mathematical model (14):

(14) $$\left.\begin{array}{c} \Psi \left(L,f\right)\leq \frac{1}{2}E_{ad}\left(P_{imp}\right)\\ T^{\prime }C=\varphi \left(\frac{1}{n}\sum_{i=1}^{n}C_{i}\left(L,t_{i}\right)\right)\\ \left\{\begin{array}{cc} \Delta T_{j+1}^{\prime }=T_{j+1}^{\prime }-T_{j}^{\prime } & \\ \Delta T_{j+2}^{\prime }=T_{j+2}^{\prime }-T_{j+1}^{\prime } & T_{j+l}^{\prime }>T_{thr}^{\prime }\\ \ldots \ldots \ldots \ldots & \Delta T_{j+l}^{`}>\Delta T_{thr}^{\prime }\\ \Delta T_{j+l}^{\prime }=T_{j+l}^{\prime }-T_{j+l-1}^{\prime } & \end{array}\right. \end{array}\right\},$$

where *Ψ* is the regularity of the acoustic field decline; *L* is the length of the acoustic measurement base; *f* is the scanning frequency of the acoustic device; $E_{ad}$ is energy potential of an acoustic device; $P_{imp}$ is pulse power of the acoustic device radiation; T′C is the room temperature; *n* is the number of reflectors in the room; *C_i_* is the measured value of the speed of sound; *t_i_* is the signal travel time along the acoustic path; $T_{thr}^{\prime }$ is the threshold value of the air temperature in the room; $\Delta T_{thr}^{\prime }$ is the threshold value of the temperature increase per second; *l* is the number of successive probings; and *j* is the number of measurement cycles by which the temperature difference (temperature gradient) is determined.

Solving the fourth task requires taking into account downward (ascending) convective movements of air in rooms with high ceilings (production facilities, warehouses, objects of high cultural, historical value, and/or religious purpose). In particular, flash and ignition in such large rooms due to convection heat transfer may not be taken into account in a timely manner, which may lead to the development of a fire. Accordingly, for high-rise buildings, it is necessary to place sensors on several levels.

Then, solving the problems of taking into account (controlling) the temperature of the air environment, and detecting fires inside the premises at a certain level *g*, will look like this:

(15) $$\left.\begin{array}{c} T^{g\;\prime }C=\varphi \left(\frac{1}{n}\sum_{i=1}^{n}C_{i}\left(L,t_{i}\right)\right)\\ \left\{\begin{array}{cc} \Delta T_{j+1}^{g\;\prime }=T_{j+1}^{g\;\prime }-T_{j}^{g\;\prime } & \\ \Delta T_{j+2}^{g\;\prime }=T_{j+2}^{g\;\prime }-T_{j+1}^{g\;\prime } & T_{j+l}^{g\;\prime }>T_{thr}^{g\;\prime }\\ \ldots \ldots \ldots \ldots & \Delta T_{j+l}^{g\;\prime }>\Delta T_{thr}^{g\;\prime }\\ \Delta T_{j+l}^{g\;\prime }=T_{j+l}^{g\;\prime }-T_{j+l-1}^{g\;\prime } & \end{array}\right. \end{array}\right\},$$

where $T^{g\;\prime }C$ is the room temperature at a certain level g; $T_{thr}^{g\;\prime \;}$ is the threshold value of the air temperature in the room at a certain level g; and $\Delta T_{thr}^{g\;\prime }$ is the threshold value of temperature increase per second at a certain level g.

Thus, taking into account the volume (height) of the room according to (15) requires transforming the mathematical model (14) into the following expression:

(16) $$\left\{\begin{array}{c} \left.\begin{array}{c} \Psi \left(L,f\right)\leq \frac{1}{2}E_{ad}\left(P_{imp}\right) \end{array}\right.\\ \left.\begin{array}{c} T^{1\;\prime }C=\varphi \left(\frac{1}{n}\sum_{i=1}^{n}C_{i}\left(L,t_{i}\right)\right)\\ \left\{\begin{array}{cc} \Delta T_{j+1}^{1\;\prime }=T_{j+1}^{1\;\prime }-T_{j}^{g\;\prime } & \\ \Delta T_{j+2}^{1\;\prime }=T_{j+2}^{1\;\prime }-T_{j+1}^{1\;\prime } & T_{j+l}^{1\;\prime }>T_{thr}^{1\;\prime }\\ \ldots \ldots \ldots \ldots & \Delta T_{j+l}^{1\;\prime }>\Delta T_{thr}^{1\;\prime }\\ \Delta T_{j+l}^{1\;\prime }=T_{j+l}^{1\;\prime }-T_{j+l-1}^{1\;\prime } & \end{array}\right. \end{array}\right\}\\ \left.\begin{array}{c} T^{2\;\prime }C=\varphi \left(\frac{1}{n}\sum_{i=1}^{n}C_{i}\left(L,t_{i}\right)\right)\\ \left\{\begin{array}{cc} \Delta T_{j+1}^{2\;\prime }=T_{j+1}^{2\;\prime }-T_{j}^{2\;\prime } & \\ \Delta T_{j+2}^{2\;\prime }=T_{j+2}^{2\;\prime }-T_{j+1}^{2\;\prime } & T_{j+l}^{2\;\prime }>T_{thr}^{2\;\prime }\\ \ldots \ldots \ldots \ldots & \Delta T_{j+l}^{2\;\prime }>\Delta T_{thr}^{g\;\prime }\\ \Delta T_{j+l}^{2\;\prime }=T_{j+l}^{2\;\prime }-T_{j+l-1}^{2\;\prime } & \end{array}\right. \end{array}\right\}\\ \ldots \ldots \ldots \ldots \\ \left.\begin{array}{c} T^{k\;\prime }C=\varphi \left(\frac{1}{n}\sum_{i=1}^{n}C_{i}\left(L,t_{i}\right)\right)\\ \left\{\begin{array}{cc} \Delta T_{j+1}^{k\;\prime }=T_{j+1}^{k\;\prime }-T_{j}^{k\;\prime } & \\ \Delta T_{j+2}^{k\;\prime }=T_{j+2}^{k\;\prime }-T_{j+1}^{k\;\prime } & T_{j+l}^{k\;\prime }>T_{thr}^{k\;\prime }\\ \ldots \ldots \ldots \ldots & \Delta T_{j+l}^{k\;\prime }>\Delta T_{thr}^{k\;\prime }\\ \Delta T_{j+l}^{k\;\prime }=T_{j+l}^{k\;\prime }-T_{j+l-1}^{k\;\prime } & \end{array}\right. \end{array}\right\} \end{array}\right.,$$

where $T^{1\;\prime }C$ is the room temperature at level 1; $T^{2\;\prime }C$ is the room temperature at level 2; $T^{k\;\prime }C$ is the room temperature at level *k*; $T^{1\prime }$ is the threshold value of the air temperature in the room at level 1; $T^{2\prime }$ is the threshold value of the air temperature in the room at level 2; $T^{k\prime }$ is the threshold value of the air temperature in the room at level *k*; $\Delta T_{thr}^{1\prime }$ is the threshold value of the temperature increase per second at level 1; $\Delta T_{thr}^{2\prime }$ is the threshold value of the temperature increase per second at level 2; $\Delta T_{thr}^{k\prime }$ is the threshold value of the temperature increase per second at level *k;* and the number of levels in the room varies from 1 to *k*.

The lower the temperature, the lower the speed of sound, and the longer it takes for the signal to travel from the emitter to the reflector and back. The higher the temperature, the higher the speed of sound, and the less time it takes for the same signal to travel from the emitter to the reflector and back. A fire indication is a temperature gradient where the temperature increases from 20 to 50 degrees in minutes (not hours).

**Figure 1:**
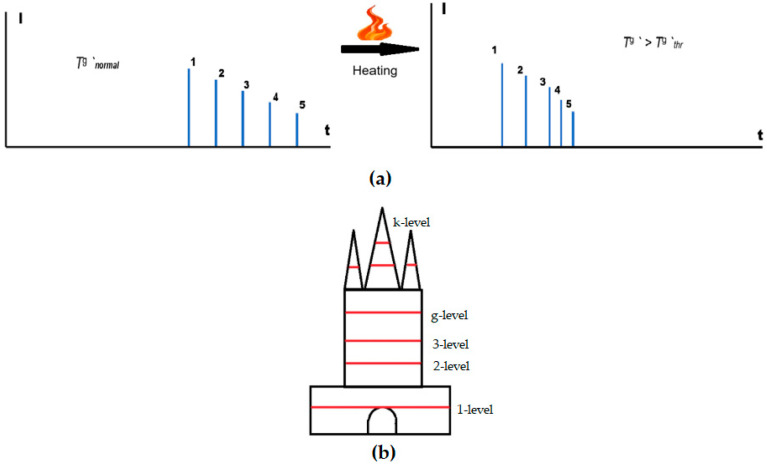
(**a**) A functional scheme of the proposed model (for the g-level), where *t* is time, *I* is the intensity of the acoustic wave, and 1, 2, 3, 4, 5 are the numbers of probing pulses; and (**b**) an illustration of the levels at which sensors can be placed in the model (based on Equation (16)).

The functioning of the model for the g-level is illustrated in Figure 1. When the temperature in the room corresponds to normal operating conditions ($T_{normal}^{g\;\prime \;}$), the passage time of probing pulses (for example, five probing pulses, as shown in Figure 1) is greatest. With an increase in air temperature ($T^{g\;\prime }$*)* in the room, the time of passage of probing pulses along acoustic paths decreases as a result of an increase in the speed of sound caused by an increase in temperature. When the temperature threshold value ($T_{thr}^{g\;\prime \;}$) is exceeded, the speed of sound increases even more, as a result of which the transit time becomes even shorter, and the interval between the reflected signals decreases.

The solutions to this mathematical model will allow the solving of a wide range of problems in both the field of fire extinguishing and safety in general: monitoring room temperature, detecting fires, monitoring humidity, moving objects into (or from) a room, monitoring the tightness of rooms and access to them, etc.

The presented model can be further implemented in various variations. However, as for most new technologies, the cost of the equipment is a limitation. According to previous studies of the applicability of acoustic technology in fire extinguishing, where the error of sound speed meters is in the range of 0.005–0.01 m/s [54], and the total error does not exceed 5% [21], one can also expect high accuracy and reliability within the framework of this model. The speed of determination, considered as the reaction time (response) of the proposed system, will be comparable to tenths of a second. The response time is the ratio of the value of the acoustic base *L* to the speed of sound propagation in the room, and is determined by the acoustic base, taking into account the direct dependence of the change in the speed of sound on temperature.

Further improvement of acoustic control systems and early detection of blockages can be achieved by combining them.

## 3. The Use of Vision Robots to Serve Humans

### 3.1. Using State-of-the-Art Techniques for Flame Detection

As shown in the work of many researchers, based on the literature review, artificial intelligence can be successfully applied for flame detection to serve humans. The use of modern techniques to apply artificial vision systems may be both a primary and a secondary means of fire detection.

Information on the use of stereovision to develop a novel instrumentation system to extract geometric characteristics from the fire front, computer vision-based methods [58,59,60], flame detection algorithms, some classifications, and edge detection techniques [61,62,63] can be found in many research papers [64,65,66,67,68,69,70,71,72,73,74,75,76]. The designed system may detect the presence of humans in addition to fire detection, with the goal of increasing the effectiveness of rescue operations aimed primarily at rescuing people. One example is the Human-like Visual Attention-based Artificial Vision (HVAAV) system, which uses machine learning to achieve artificial human-like visual attention [77]. By hybridizing models, it becomes possible to tune the parameters of the system to human perception of vision, which can influence emergency services, as well as mobile robots that extinguish flames, to take proper action.

In practice, many fire pixel detection techniques are used, as well as color models. The algorithms used often use color rules in different color spaces (RGB, YCbCr, HSI, HSV, YUV, L*a*b*) or combinations of these. The efficiency of the rules varies depending on the type of fire pixel. Therefore, the selection of appropriate rules is a problematic issue.

A detailed consideration of this topic, including saliency detection (saliency maps), segmentation, and extraction of salient objects, is presented in [77], including a description of the model, visual attention, human-like visual attention through the human ‘Eye Fixation’ paradigm, a Genetic Algorithm (GA)-like tuning process, data filtering, and, finally, interpretation of the visual attention map. Fire pixels can be classified based on fire color and the presence of smoke, while non-fire pixels can be classified based on average image intensity. Such robots can be used in crisis management, which is outlined in this paper.

It becomes possible to design a system based on artificial vision systems using mobile robots. These robots can be wheeled robots, as well as robots for special tasks, allowing one to extinguish flames. Research [77] shows an example and evaluation of such a system. It was implemented with the use of a mobile robot from NEXTER Robotics (Wifibot-M). It may be controlled both by wireless (Wi-Fi) and wired (Ethernet) methods. The software driver is dedicated to the Windows operating system, allowing the robot to be controlled with a joystick or using a virtual simulation. Camera images can be captured and then collected using the AXIS Encoder web interface or, for example, the dedicated AXIS Camera Client application.

In practice, using artificial intelligence to detect flames, networks learn from static images or video streams [78,79,80,81,82,83]. This requires a computer module, a camera, and a powerful image processing unit. Many processing systems may be applied for network implementation (e.g., CPU, GPU, VPU). The controller can generate a variety of video signals. Such an intelligent sensor does not need to be expensive. Hardware could be based on a Raspberry Pi board, which allows for the support of multiple operating systems. In software, many libraries, such as OpenCV 4.11, Matplotlib 3.10, TensorFlow 2.0, Imutils 0.5, and NumPy 2.2, may be applied. The system can be standalone, or it may work on other hardware platforms. In addition, it is possible to integrate it with an acoustic extinguisher using relay modules (24 V control signals), which can enable (if flames are identified) a discrete activation of the extinguisher, without the need for additional sensors. For fire sensors and fire detection systems that utilize cameras and machine vision, various machine learning algorithms for fire detection and different types of interface can be applied for data transmission. When sensors have built-in data processing and fire detection capabilities, widely used communication interfaces, such as RS485, Modbus, or building automation protocols such as BACnet or KNX, can be employed. In cases where a larger amount of data needs to be exchanged, interfaces such as Ethernet or Wi-Fi can be applied. If sensors or sensor systems need to operate wirelessly, protocols such as LoRa or NB-IoT can be utilized. A case study of the use of neural networks for flame detection, supported by examples, is provided in the next part of the paper.

### 3.2. Fire Detection Techniques Using Artificial Vision Systems and Video Cameras

As stated previously, an acoustic fire extinguisher may be equipped with a smart sensor. Research on this topic is being conducted, which is a scientific novelty. In this context, research and achievements in terms of fire detection are essential.

In practice, since 2000, intensive research has been conducted on the feasibility of using computer vision and cameras to detect fires, especially in uninhabited areas and in wildlife (e.g., [60,78]). An overview of the performance of pixel detection algorithms for these cases can be found in the works [60,84,85]. Depending on the environmental conditions (unstructured or structured), the results are different. For example, the colors yellow, orange, and red dominate in intervals in outdoor fires. Green, yellow, and brown appear in outdoor experiments (scenes are unstructured). Multiple color spaces and their combinations can be applied for detection, which will be the subject of the next section [73,74,75,76,84,85,86,87,88,89,90,91].

The resulting set may be used to compute functions and constants for the rules, as well as for pixel training [71]. After training, each pixel can be assigned a probability that indicates to what extent the pixel is part of the flame. Given an appropriately chosen threshold, this results in candidate pixels for further processing [92]. The important feature is the percentage of fire pixels in a particular image, which is obtained by dividing the number of fire pixels by the total number of all image pixels. The fire pixels detected in this way are further processed based on other features. Important features are those that deal with geometric information, such as the degree of spread, height, and slope of the flames [71,93,94,95]. Indeed, not only color properties (flame pixels) [66,78,86], but also shape irregularity [96], texture [97], and even dynamics [68,98] are useful for flame detection. To make flame detection more efficient, some features can be combined (e.g., [92,99]), where the pixel detection criterion, as well as methodology, are usually tested with isolated parameters [59]. In addition, hybrid flame detection frameworks and probabilistic methods are used to improve detection performance [92,100,101,102]. From the point of view of firefighting action, the rapid extinguishment of flames is crucial [103,104]. The infrared band can also be applied for data analysis [38,105,106]. Table 1 lists the references cited and the properties they consider for fire detection techniques [46].

In general, none of the imaging techniques are fully universal, which means that they are not adapted to segmenting all fire scenarios, and thus their performance will not always be the same. A problematic issue is the lack of a universal fire imaging data set that would be universally accepted as a benchmark and used to test different methods. Only then would it be possible to reliably compare the results obtained with their use. Such images may be applied to analyze fusion algorithms, the performance of fire segmentation processes, or the use of motion, as shown in [58,59,77]. To estimate the performance of the algorithms, benchmarking can be used. Since color segmentation is generally the first step in the fire segmentation process, the selection of an appropriate algorithm is crucial [60].

### 3.3. Fire Detection Categories

In the last decade, computer vision has started to be used for effective fire detection [39,40,107,108], early fire extinguishment [109], fire measurement, and prediction of fire behavior [110,111]. The most important step in computer vision analysis is the detection of fire pixels, as this determines the accuracy of further processing.

In practice, there are several categories of fire detection methods. Some use color rules, while others are based on machine learning. In the second case, the learning is performed on a data set that contains fire pixels and non-fire pixels. Since it is a key issue that the database includes heterogeneous color images, they are selected to capture as many different flame contexts as possible in a heterogeneous environment. The performance of each method varies because it depends on many factors, such as the color of the fire, the type of fuel, the presence of smoke, and the environmental conditions [60].

Color-based salience detection can be implemented with binary models and exponential functions [61]. In addition to color and motion detection, among other things, edge detection, created on the basis of mathematical models and the resulting algorithmic solutions, is relevant in this field. In general, it is possible to process a specific feature in a separate scheme, an example of which is given in [62]. On the basis of the results presented there, it was shown that it is problematic to select a detector that has the same efficacy in many cases. Mathematical methods known from security sciences can be used for forecasting [112,113,114,115]. In practice, the features used, together with machine learning classifiers and artificial intelligence, allow for efficient fire detection (the subject of scientific effort is to reduce the false detection rate to a minimum).

A scientific innovation made in recent years has been the combination of image processing techniques or dedicated expert systems that can support human recognition abilities, but none of these approaches has been fully implemented as an embedded solution [61,63,64,65]. Therefore, the authors decided to try to fill the gap in this area. From a practical point of view, fire segmentation algorithms apply color criteria in different color spaces [116]. To estimate the performance of different segmentation methods, standard metrics can be used, whose role is to compare segmented images with manually segmented images [60]. Many performance metrics are helpful for this purpose, such as the Matthews Correlation Coefficient [69], F-score [70,71], and the Hafiane Quality Index [72]. Machine learning involving logistic regression generally obtains the best performance (the highest robustness against smoke and color changes is reported). Therefore, the results achieved by the logistic regression method for most categories are the best [71]. This includes determining fire pixels that contain smoke and fire pixels without smoke (the support vector is useful for classification) [60]. A popular approach is fire detection using Bayes’ theorem, which allows for multiple representations of conditional probabilities. This method may be applied without a prior learning step. In practice, a measurement vector can be formed, for example, based on texture (according to the entropy of the fire pixels, one checks whether the fire is textured), combinations of different color spaces, and a multidimensional Gaussian distribution (the ratio of probabilities a priori for fire and non-fire classes can be changed, depending on the given conditions) [60].

### 3.4. Pixel Detection Methods

In practice, two basic types of models can be distinguished: empirical models with experimental thresholds [66,68,78], and statistical models [67,68] that use real data for training (see Table 2).

For flame detection, the number of images is important. The information they contain is crucial for the choice of color space and for rules for the detection of fire pixels. In practice, the more images, the better the color space and fire pixel detection rules that may be chosen [71]. There are many algorithms for fire detection. For example, for the visible spectrum, the algorithms applied often use rules in different color spaces (RGB, YCbCr, HSI, HSV, YUV, L*a*b*) or combinations of these. Application examples of color spaces used in roles in the visible spectrum are listed in Table 3. From a practical point of view, each pixel detection method is a logical combination of rules that contain basic mathematical operations. The paper [71] discusses, in detail, the issues of expert systems, majority voting, and machine learning, specifically logistic regression, using rules as features to combine rules. It is difficult to compare the performance of the color segmentation techniques used, as it depends on the category of the image. Factors such as lighting, dominant color, or the presence of smoke translate into the results achieved. In general, lighting conditions can affect the natural colors of fires. Therefore, the choice of algorithm in an operational scenario depends on many factors. Since RGB models are sensitive to luminance changes [68,73,74,75,76,84], it is common to apply chromatic models in YCbCr [66,85] and HSV [86,87], or other [88,89,90] spaces. This means that fire pixels are marked with one of the available colors in a certain color space. The dominant color is always the one that covers most of the fire pixels. The work [74] describes a method based on supervised learning in the RGB color space that is resistant to smoke. The segmentation presented in [84] gives good results for daytime fires (without smoke). When using the YCbCr color space, good results were obtained for orange pixels [66,85]. Similar results were achieved when using the L*a*b* space [89]. In practice, it is possible to use combinations of different color spaces, which allows increased effectiveness of the techniques applied, depending on the color of the pixels (examples for orange and yellow pixels are given in works [78,107], while for fire pixels without smoke, examples are given in [91]). In general, tests performed using real images include flame detection (the most common case). Smoke detection or wildfire detection is also possible [71,105,106,107,117]. The method described in [116] works well when the intensity of the environment changes. In turn, in [71], a technique applying logistic regression was used, which turned out to be robust against changes in smoke and color. There, a combination (majority voting and logistic regression) was described. In practice, Bayesian segmentation is robust against fire color changes [60]. It is worth highlighting that combinations of different color spaces may be applied [91], which is a commonly used method.

**Figure 2:**
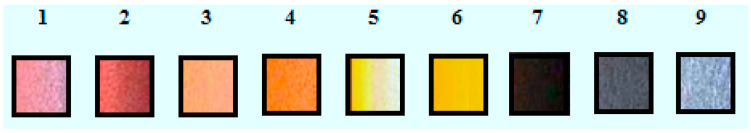
Examples of pixels used for pixel learning. The following categories may be listed: 1—red with smoke; 2—red with no smoke; 3—orange with smoke; 4—orange with no smoke; 5—yellow-white with smoke; 6—yellow-white with no smoke; and pixels: 7—low brightness; 8—medium brightness; and 9—high brightness. The RGB values of the pixels corresponding to the colors of fires can vary across different systems.

As previously mentioned, firstly, the crucial operation is the detection of fire pixels, as it affects the accuracy of further data processing [59]. The fire emits radiation in a wide spectral band (the color may change, and it may have different luminance, depending on the background and brightness). The environment affects the image, as do the physical conditions; a prime example is smoke, which can mask the fire region. In practice, the percentage of fire pixels overlaid with smoke is determined with pixel classification (into pixels with and without smoke), performed using a support vector machine [59,71]. In practice, the rules are analyzed by means of pixel categories, which are created on the basis of a specific classification, including both fire pixels and non-fire pixels. To build a learning set of pixels, the fire pixels are classified into several categories, depending on the color and the presence of smoke [71]. In turn, non-fire pixels are classified into several brightness levels. These categories are subjected to additional classification, an example of which is given in Figure 2. In this case, there are some basic categories depending on the color (red, orange, yellow-white) and the presence of smoke (smoke is present or not). Non-fire pixels that have high brightness represent the color of the sky and smoke. In turn, a green color is most often present for pixels with low and medium brightness, suggesting that their source is the plants seen in the images [59].

These colors demonstrate the pixel colors used for fire detection. Their mapping to a specific color palette or to certain RGB values is performed within the corresponding vision system, along with the necessary adjustments for brightness and contrast of the images obtained from the vision sensor. Within each category, individual pixels are represented by a feature vector that is created based on color features (from different color spaces). The average feature vector is calculated for each category. Pixels are sorted according to the distance of their vectors from the average feature vector. Then, they are sampled uniformly, resulting in a desired number for each category. For ease of processing, learning pixels can be arranged in such a way that an image is formed whose individual parts correspond to the pixels. The results depend on the category of pixels. From this, it can be deduced that, depending on the color, the presence of smoke, or the intensity, different rules for different color spaces give different results. For example, the rules proposed by T. Toulouse, L. Rossi, T. Çelik, and M. Akhloufi are more effective for fire pixels without smoke (especially for orange pixels) [71]. Their paper provides a comparative analysis of state-of-the-art fire color detection techniques in the context of geometric measurements. The methods described are effective in many categories. A comparison of the rules can be found there (the results are compared) [71].

The paper [92] describes an autonomous method for flame detection in video recordings. For this purpose, a detection system based on the properties of color, dynamic range, texture, and flame flickering is proposed, which is realized by analyzing the variations in pixels in the wavelet domain. The accuracy obtained exceeds 95%, which is a good result. For this purpose, a hybrid flame detection system is suggested [92]. The color distribution is modeled by a Gaussian Mixture Model with an estimated number of Gaussian components obtained by a Dirichlet process; thus, it avoids the deviations that result from an improper number of Gaussians that were experimentally determined. An incorrect number of components translates into a poor estimate of the means and covariance matrix, which in turn affects the estimate of the flame color distribution. Therefore, a hybrid flame detection framework based on the Dirichlet Process–Gaussian Mixture improves the detection performance [92,100,101]. The flame color model based on this approach is processed together with saturation analysis and wavelet transform. The method proposed in [92] can detect most of the fire pixels, including those behind the smoke. Although the model omits some pixels from the inner part of the flame, it should be noted that the successfully detected contour fire pixels better reflect the dynamic properties of the flame [92].

On the basis of this analysis, it can be concluded that no technique is universal (not equally effective for segmenting all fire scenarios). The combination of multiple rules, using different color spaces, allows one to obtain a better result compared to the result achieved with the use of individual rules. Therefore, the main difficulty is related to the selection of appropriate rules, which are created depending on the type of fire pixels, as they do not always have the same efficiency for all types of fire pixels. The difficulty comes down to choosing the proper rules to combine and assigning them the appropriate weights. Some methods combine rules with the same weight, while others use machine learning when all rules are input rules. The effectiveness of combining individual rules and methods depends on the category (as stated earlier, no single rule can perform equally well in all categories). The image category affects the implementation of color segmentation (e.g., color, smoke, etc.). Therefore, depending on these factors, different results are obtained for the processed data set images. Since the images from the data set are characterized on the basis of fire, color, brightness, and smoke, it becomes possible to determine the usefulness of a particular algorithm. In turn, information on additive networks can be found in [118]. Some approaches require significant computational power and do not operate in real time, which is critical when rapid flame suppression is needed [102,103,104]. It is important that the scaling time does not exceed 1 s, and that the distance between the camera and the flame location is small [77]. Information on the classification of flame extinction based on acoustic oscillations using artificial intelligence methods can be found in [33]. In practice, acoustic fire extinguishers may provide an additional means of fire protection, and can be permanently installed in areas exposed to fire or be mobile fire protection devices installed, e.g., in robots capable of visual image processing. It is worth noting that emergency management expenditures are increasing, resulting in increased interest in the use of robots for various purposes [119,120]. In the case of adverse events, crisis management plays a key role [121,122], and communication is then often carried out using a variety of wireless technologies, especially satellite telecommunications [123,124,125], whereby the quality of signals, depending on their type, is determined by a variety of factors [126,127,128,129,130,131]. The spectrum of service integration translates into a change in the way in which fire risks are identified and assessed, as well as controlled [132,133,134].

## 4. Unresolved Problems

For firefighting operations, modern technologies based on artificial vision systems implemented in robots may support the work of firefighters. In general, the behavior of a given system can be adapted to the firefighter’s way of looking. The benefit of such a solution is to obtain artificial visual accuracy, which may help firefighters to assess intervention conditions or rescue operations [77]. An image that has one distinct flame is considered simpler for vision processing than an image containing several flames. Due to the perception of the human eye, complex or ambiguous images may or may not attract the attention of a potential human. This is important because during a fire, there is a need for firefighting and rescue operations, especially where they are most desired and urgent. This solution is particularly applicable to sites and objects that are far from human concentrations, in forests, taiga areas, and sparsely populated areas. However, such an environment limits the application of some flame suppression technologies.

**Figure 3:**
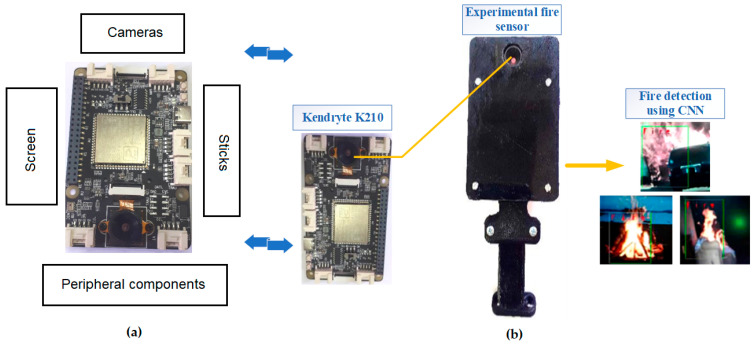
(**a**) An example of the hardware of the system (overview); and (**b**) an experimental sensor for fire detection developed by the authors.

In practice, as previously mentioned, many types of communication can then be used, including free-space optical interconnects. It is also important to take into account specific propagation conditions, depending on the frequency band used for communication [135,136]. In the long term, further work can be expected to increase the range of intelligent acoustic extinguishers, which, equipped with an intelligent module, do not require human presence [34,35,36,37,38,45]. An example of a system hardware based on edge computing AI HAT and built with a Kendryte processor is provided in [137]. It is a powerful and inexpensive artificial intelligence platform that can be applied as an independent board. It is equipped with a neural network processor and a dual-core 64-bit CPU. In addition, the board has several peripherals, including PWM/SPI/I2C GPIO, an LCD screen, and camera interfaces. It can be applied to implement deep neural networks to detect flames. A detailed description of vision fire sensors can be found in publications [34,35,36,37,45], amongst others. Such systems detect firebreaks, and could be connected to acoustic fire extinguishers. Figure 3 shows an example of a solution that can be applied to the design of a fire detection system. In the range of practical implementation, the left object is the sensor, and the right object is the electronic board inside it. In the processor, a neural network that can detect fires is inserted (Figure 3b).

Moreover, it is important to study the extinguishing capabilities of acoustic waves emitted from sound sources [39,40,41,42,43,44,45,46,47,48,138,139,140,141,142]. Since this technology has not yet been fully explored, research is being conducted on both the use of modulated and unmodulated waves to extinguish flames, depending on the class of fire [21,38,41,45,46,47,48,140,143,144,145,146]. Apart from the work of the authors and a few others, most of which are included in the bibliography of this article, there is a lack of research conducted in this area, especially using waves emitted by sound sources with high and very high acoustic power. Further work aimed at using firefighting drones in the initial stage of a fire can also be expected [147], which is a promising research direction. A separate group is conceptual work and research to learn about new flame-retardant materials [148,149,150,151,152,153,154], from which, e.g., components of fire extinguishers may be built [13].

Since visible-band flames can be detected using infrared, images may be processed in infrared. Then, a much higher intensity level of the fire pixels compared to other pixels is observed [107]. In this case, it is crucial to determine an appropriate threshold at which it becomes possible to distinguish fire pixels from background pixels. However, false alarms can be caused by cloudiness during infrared processing (clouds may be in the image) [59], which is important for detecting wildfires, but does not apply to indoor detection. Non-fire pixels that have high brightness refer to the color of the sky and smoke. Problematically, processed images can have fire-like areas corresponding to hot gasses [59]. Since it is easier to detect a fiery pixel in infrared images, modern algorithms are often developed using image fusion. Performance comparisons between different algorithms based on image fusion or pixel motion are then performed, based on commonly known criteria. More research is needed in this area to improve efficiency.

The social impact of fire detection using machine vision sensors and systems is immense. These systems not only enable the automated detection of fires in forests, agricultural areas, and populated locations, but also significantly enhance the efforts of firefighters in preventing the occurrence and escalation of large, life-threatening fires. The use of such systems ensures public safety, helps to protect citizens’ property and lives, and provides an added layer of security. The use of acoustic technologies in fire extinguishing is promising. At the same time, the issue of their practical implementation remains open. The costs of building and testing complete equipment are quite high; however, with sufficient interest from businesses and implementation in production, these costs can be expected to decrease. The next issue that will need to be addressed at the national (international) level is the regulatory framework for the use of acoustic technologies in fire extinguishing, including with the use of other systems (artificial intelligence, artificial vision, etc.). The positive aspects of using such technology include the absence of the need for additional costs for consumables (as when using water and fire extinguishing agents) when using it, environmental friendliness, and the possibility of combining it with other subsystems, as well as the preservation of cultural, historical, and religious sites and valuables when using this technology in practice. Although the cost of some fire detection systems that utilize cutting-edge technologies may be high, their deployment ensures that fires with destruction far exceeding the systems’ value are prevented.

## 5. Conclusions

The acoustic method can be considered as an alternative to known methods of preventing and extinguishing fires of liquid, solid, and gaseous substances; however, it requires further research. Undoubtedly, the use of artificial intelligence, by integrating vision systems and cameras, makes it possible to detect flames not only in open areas, but also in closed spaces, which is particularly important when the use of classical sensors is severely limited or impossible.

This paper proposes a mathematical model of acoustic temperature control in a local room and the detection of fires and outbreaks inside it by means of pulse acoustic probing. This model is a system of three dependencies. The first dependency makes it possible to calculate and determine the main technical parameters of the designed acoustic devices for rooms with known dimensions and, conversely, to calculate the dimensions of rooms that can be serviced by specific devices. The second dependency allows one to determine the average air temperature in the room, taking into account the measurements taken. The third dependency, due to the exceeding of the threshold value of the air temperature in the room and of the threshold value of the temperature increase per second, ensures the detection of fires and outbreaks inside the room. Taking into account the volume (height) in large rooms is ensured by introducing a certain number of temperature control levels for the second and third dependences. It is expected that the implementation of the developed model will allow for both the creation a new generation of devices and instruments for preventing fires inside the premises, and the modernization of existing fire alarm systems of premises, as well as the solving of a wide range of problems in the field of security, including the protection of objects of high cultural, historical, and religious value.

Notably, visual relevance plays an important role in gaining a better awareness of the environment under study. Computer vision makes it possible to detect flames and predict the behavior of a fire. The effective and fast detection of flames allows one to reduce the losses caused by fire. For the purposes of firefighting actions, modern technologies based on artificial vision systems can be used, which may be implemented in robots or be part of the firefighting system, e.g., using environmentally friendly acoustic technology on the basis of European cooperation, which is specially designed for indoor use. Such systems can work together as a standalone platform, or they can cooperate with other modules. In practice, artificial intelligence may be applied in industrial applications, such as production lines, where it is possible to use acoustic flame extinguishing technology in a simple way. Fire detection sensors can be integrated into smart city infrastructures by connecting them to Internet of Things networks for real-time monitoring and data sharing, enabling faster fire response. These sensors can be connected with building automation systems of smart buildings to coordinate emergency actions in the buildings. Fire detection systems can also be integrated with emergency response systems to provide precise location data and improve coordination during fire emergencies. Additionally, artificial intelligence algorithms can enhance fire detection and optimize evacuation plans, while public notification systems can alert citizens in real time. Therefore, further research related to eco-friendly acoustic firefighting and flame detection using artificial intelligence is expected. This includes new work aimed at improving flame detection methods using visible- and infrared-band cameras and enhancing the effectiveness of the algorithms applied (more than 90–95%).

The development and implementation of acoustic fire extinguishing systems using artificial intelligence and computer vision is expected to contribute to the prevention (early detection) of fires, which in turn will improve the environmental, economic, and social development of individual industries, regions, and countries.

## Figures and Tables

**Table 1:** Properties for fire detection techniques.

Properties	List of Publications
Color	[66,78,86]
Texture	[97,99]
Shape	[87,96,103]
Flickering properties	[68,92]
Dynamics	[68,86,97,98]
Combined features	[61,62,63,64,99]
Processing techniques	

**Table 2:** Two basic types of models.

Type of Model	Description
Empirical inequality models	The use of these models is based on empirical inequality (there are experimental thresholds). They work well in detecting real flame pixels, but not in filtering out noise.
Statistical models	The use of these models is based on models trained with real data. The effectiveness is higher if an appropriate model trained with sufficient data is used (the number of mixture components is not known in advance).

**Table 3:** Application examples of color spaces used in roles.

Color Space Used for Detection	Authors	List of Publications
RGB	W. Phillips III, M. Shah, and N. da Vitoria Lobo; B. U. Töreyin, Y. Dedeoğlu, U. Güdükbay, and A. E. Çetin; T. Çelik, H. Demirel, H. Ozkaramanli, and M. Uyguroglu; B. C. Ko, K-H. Cheong, and J-Y. Nam; J-F. Collumeau, H. Laurent, A. Hafiane, and K. Chetehouna	[68,74,75,76,84]
YCbCr	T. Toulouse, L. Rossi, M. Akhloufi, T. Çelik, and X. Maldague; T. Çelik and H. Demirel	[60,66]
HIS	W-B. Horng, J-W. Peng, and C-Y. Chen	[90]
HSV	C-B. Liu and N. Ahuja	[87]
YUV	G. Marbach, M. Loepfe, and T. Brupbacher	[88]
L*a*b*	T. Çelik; A. Z. Chitade and S. K. Katiyar	[89,116]
Other, combinations of color spaces, roles	T. Chen, P. Wu, and Y. Chiou; J. Chen, Y. He, and J. Wang; L. Rossi, M. Akhloufi, and Y. Tison; S. Rudz, K. Chetehouna, A. Hafiane, H. Laurent, and O. Séro-Guillaume	[58,78,85,91,117]

## Data Availability

Not applicable.
